# Dabrafenib-Trametinib and Radiotherapy for Oligoprogressive *BRAF* Mutant Advanced Melanoma

**DOI:** 10.3390/biomedicines11020394

**Published:** 2023-01-29

**Authors:** Ernesto Rossi, Giovanni Schinzari, Francesco Cellini, Mario Balducci, Mariangela Pasqualoni, Brigida Anna Maiorano, Bruno Fionda, Silvia Longo, Francesco Deodato, Alessandro Di Stefani, Ketty Peris, Maria Antonietta Gambacorta, Giampaolo Tortora

**Affiliations:** 1 Medical Oncology, Fondazione Policlinico Universitario Agostino Gemelli IRCCS, 00168 Rome, Italy; 2 Medical Oncology, Università Cattolica del Sacro Cuore, 00168 Rome, Italy; 3 Radioterapia Oncologica ed Ematologia, Dipartimento di Diagnostica per Immagini, Fondazione Policlinico Universitario Agostino Gemelli IRCCS, 00168 Rome, Italy; 4 Radioterapia Oncologica ed Ematologia, Dipartimento Universitario Diagnostica per Immagini, Università Cattolica del Sacro Cuore, 00168 Rome, Italy; 5 Oncology Unit, IRCCS Foundation Casa Sollievo della Sofferenza, 71013 San Giovanni Rotondo, Italy; 6 UOC Radioterapia Oncologica Molise ART, Gemelli Molise Hospital, 86100 Campobasso, Italy; 7 Dermatology, Fondazione Policlinico Universitario Agostino Gemelli IRCCS, 00168 Rome, Italy; 8 Dermatology, Università Cattolica del Sacro Cuore, 00168 Rome, Italy

**Keywords:** metastatic melanoma, BRAF inhibitors, MEK inhibitors, radiotherapy, case series, BRAF, dabrafenib, trametinib, target therapy, beyond progression

## Abstract

The clinical management of metastatic melanoma has been changed by BRAF (BRAFi) and MEK inhibitors (MEKi), which represent a standard treatment for *BRAF*-mutant melanoma. In oligoprogressive melanoma patients with *BRAF* mutations, target therapy can be combined with loco-regional radiotherapy (RT). However, the association of BRAF/MEK inhibitors and RT needs to be carefully monitored for potential increased toxicity. Despite the availability of some reports regarding the tolerability of RT + target therapy, data on simultaneous RT and BRAFi/MEKi are limited and mostly focused on the BRAFi vemurafenib. Here, we report a series of metastatic melanoma patients who received fractioned RT regimens for oligoprogressive disease in combination with the BRAFi dabrafenib and the MEKi trametinib, which have continued beyond progression. None of the cases developed relevant adverse events while receiving RT or interrupted dabrafenib and trametinib administration. These cases suggest that a long period of dabrafenib/trametinib interruption during radiotherapy for oligoprogressive disease can be avoided. Prospective trials are warranted to assess the efficacy and safety of the contemporary administration of BRAF/MEK inhibitors and radiotherapy for oligoprogressive disease.

## 1. Introduction

Melanoma represents one of the most frequent solid tumors in Europe and the US, most commonly presenting as cutaneous melanoma [1]. In Europe, the incidence of cutaneous melanoma is 9 new cases/100,000 person/year, the mortality 2-3/100,000/year [2]. In the US, melanoma is one of the most common cancers in people younger than 30 years [3]. The non-cutaneous subtypes of melanoma, such as mucosal, acral, uveal, and conjunctival melanoma, occur less frequently [4].

Around 50% of melanomas harbor mutations in the *BRAF* gene. Among them, *V600E* is the most common (detected in 70% of cases), followed by *V600K* (20%) and, more rarely, other uncommon mutations (such as *V600D/R* or non-*V600* mutations) [5,6]. Among the other subtypes of melanoma, conjunctival melanoma has similar rates of *BRAF* mutations similar to cutaneous melanoma. On the contrary, *BRAF* mutations are more rarely detected in acral melanoma (10–15% of total), mucosal melanomas (5% of cases), and uveal melanoma [7]. *BRAF* mutations play a predictive role in melanoma therapy [8]. In fact, the combination of BRAF inhibitors (BRAFi) and MEK inhibitors (MEKi) represents a standard treatment for *BRAF*-mutant melanomas [9,10].

Historically, melanoma is known for its poor sensitivity to radiation therapy [11]. Nevertheless, radiotherapy (RT) is usually administered at various metastatic sites, mostly with palliative intent, often obtaining satisfactory results in terms of local disease control [8,12]. However, when RT is administered while target therapy (TT) is ongoing, the interactions need to be carefully monitored because of potentially increased toxicity, especially in the case of high doses of RT [13]. BRAFi and MEKi potentially interact with radiation and may be associated with increased toxicity in the lung, skin, central nervous system, liver, and other visceral organs [14,15]. Data regarding the administration of RT while TT treatment is ongoing are poor [16]. The Eastern Cooperative Oncology Group (ECOG) suggests the interruption of these inhibitors during RT administration [17]. It is advisable to hold these drugs at least 3 days before and after RT administration in the case of fractioned regimens and 1 day before and after in the case of stereotactic doses [13,17]. However, the reports are retrospective, and prospective data are still lacking. High doses of RT seem to be associated with higher toxicity than fractioned regimens or stereotactic radiotherapy (SRT) [13]. On the other hand, the same radio-sensitizing effect of target agents could increase the efficacy and maximize the effect of radiotherapy [18,19]. Therefore, the challenge of interrupting or continuing TT during RT needs to be addressed in larger studies.

Hereinafter, we report a series of 5 Caucasian metastatic melanoma patients (Table 1), who received fractioned RT regimens in different sites beyond progression and during the treatment with dabrafenib (BRAF inhibitor) and trametinib (MEK inhibitor).

## 2. Case Series

### 2.1. Case 1

**Figure 1:**
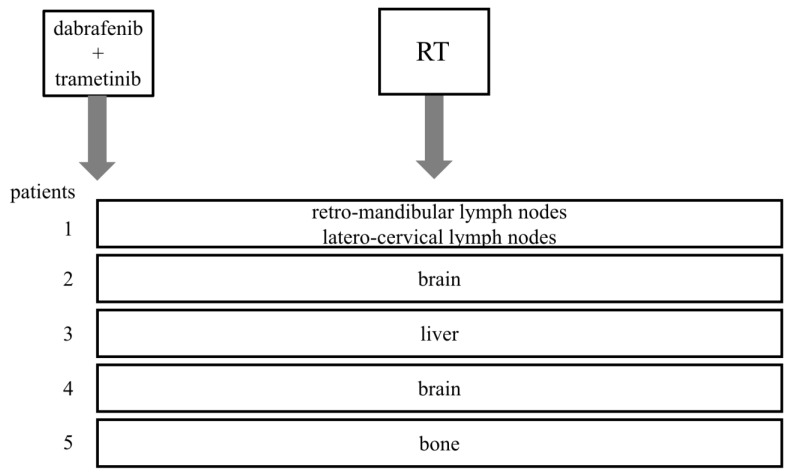
Sites of progression treated with radiotherapy (RT) during treatment with dabrafenib + trametinib.

The first report regards a 70-year-old patient with *BRAF*-mutant conjunctival melanoma. In April 2016, he underwent a complete excisional biopsy of the left bulbar conjunctiva, with evidence of a conjunctival melanoma (thickness of 0.3 cm), *V600 BRAF* mutant. In July 2017, after the appearance of swelling of the right parotid region of about 20 mm in diameter, an 18-fluoro-desossi-glucose (FDG) positron emission tomography (PET)/computerized tomography (CT) scan was performed, showing metastases in the right parotid gland and latero-cervical lymph nodes. A right parotidectomy with latero-cervical lymph node dissection was performed, and the histologic exam confirmed the presence of metastases from conjunctival melanoma in the parotid gland and in 1/13 lymph nodes. A V600E mutation of the *BRAF* gene was detected. The post-operatory 18FDG PET scan revealed a residual tumor in the retro-mandibular and latero-cervical regions (maximal SUV 3.9) and a metastatic nodule in the right hypogastric mesenteric tissue. Dabrafenib (150 mg twice daily [TD]) and trametinib (2 mg once daily [OD]) were started to achieve a partial response. In April 2019, radiological assessment showed a progression of disease on the right retro-mandibular lymph nodes. Therefore, the patient underwent RT on the only progressive site (2000 cGy), continuing treatment with dabrafenib and trametinib (Figure 1). In January 2020, an increase of left latero-cervical lymph nodes occurred, without progression of disease at other sites. Radiation therapy on left latero-cervical lymph nodes was performed (2000 cGy), without interrupting TT. No adverse events (AEs) were reported during concomitant RT and TT administrations. The patient is still on treatment.

### 2.2. Case 2

A 70-year-old man with a history of cutaneous melanoma (right scapular region, Breslow 5 mm, rare tumor infiltrating lymphocytes (TILs), evidence of regression, and satellite lesions; pT4b, sentinel node negative) underwent a complete node dissection of the right axillary region for the appearance of macroscopic lymph node metastasis. Histological examination revealed melanoma metastases in 8/15 lymph nodes. A *BRAF V600E* mutation was found. The 18FDG PET/CT scan detected metastases in the lungs, abdominal lymph nodes, and adrenal glands, with normal LDH. In March 2019, therapy with dabrafenib (150 mg twice daily) and trametinib (2 mg daily) was started. The patient achieved a partial response after 3 months. However, after 6 months of therapy, brain magnetic resonance imaging (MRI) showed 3 brain metastases in the left orbito-frontal area (11 and 7 mm) and in the right cerebellum (7 mm). Moreover, colonoscopy revealed a stenosing and bleeding mass located 60 cm above the anus. The patient received SRT for brain metastases with a total dose of 2550 cGy per lesion, delivered in 5 daily fractions. Therapy with dabrafenib and trametinib was never interrupted during RT, and no AEs were reported. One month after SRT, the patient underwent a partial resection of the right colon, with evidence of metastasis of melanoma in the anal nodule, 4/5 lymph nodes, and mesenteric micro-nodules. Overall, the patient was on treatment with dabrafenib and trametinib for 14 months.

### 2.3. Case 3

A 65-year-old patient with a history of cutaneous melanoma (thickness 1.1 mm, sentinel lymph node biopsy—SLNB-negative for metastasis) came to our attention in February 2019 with a tumefaction in the right axilla. The 18FDG PET/CT scan showed an uptake of a lymph node in the right axilla (diameter 38 mm), many subcutaneous lesions in the retro-pectoral region and right chest, a liver metastasis of the VII-VI segment (maximum diameter 69 mm), and three lung metastases. A *BRAF V600E* mutation was found in the primary lesion. Dabrafenib and trametinib at the standard dose were started, and after the first month of therapy, nodosus erythema developed on the right limb and was treated with continued corticosteroid therapy. Neither dose delay nor dose reduction was necessary for TT. The following 18FDG PET/CT scans after three and six months of therapy documented the reduction of all the lesions, except for lymphadenopathy in the right axilla (increased up to 68 mm). In January 2020, the patient underwent radial axillary lymph node dissection. After 9 months of therapy, the 18FDG PET/CT scan evidenced a dimensional increase in liver metastasis (maximum diameter 99 mm). After a multidisciplinary discussion, the patient underwent a loco-regional radiant treatment (total dose 3000 cGy, delivered in 3 fractions). Therapy with dabrafenib and trametinib was continued without any clinical or laboratory evidence of toxicity. Moreover, the 18FDG PET/CT scan after RT showed shrinkage of the hepatic lesion (decreased to 48 mm). No adverse events occurred during contemporary RT and TT administration.

### 2.4. Case 4

A 47-year-old patient presented in January 2018 with a pigmented and ulcerated lesion of about 1 cm located in the right subscapular area. There were no relevant comorbidities or concomitant medications. The patient underwent a complete excisional biopsy of the pigmented lesion. Histological analysis demonstrated cutaneous melanoma. However, a post-operatory CT scan showed the presence of a nodule in the right lung (7 mm) and multiple lymph node metastases in the right axillary region (the major of about 16 mm). The patient underwent a complete right axillary lymph node excision, with histologic evidence of metastases of cutaneous melanoma. A radiological assessment was repeated, and a CT scan showed an increase in lung metastasis (12 versus 7 mm). A resection of the lung metastasis was performed. A *BRAF V600E* mutation was detected. A new CT scan showed the presence of multiple lung and brain metastases. Whole brain RT was performed (total dose 2000 cGy). In November 2018, therapy with dabrafenib (150 mg TD) plus trametinib (2 mg OD) was started; however, in July 2019, the CT scan showed the presence of multiple brain metastasis, and the patient underwent a whole brain RT (WB-RT) (total dose 1200 cGy, delivered in 10 fractions of 120 cGy TD). Therapy with dabrafenib and trametinib was continued during RT without AEs. No dose reduction was required.

### 2.5. Case 5

In 2019, a 52-year-old patient with a history of cutaneous melanoma (Breslow 3.2 mm, Clark IV, ulcerated, moderate TILs, absence of regression and vascular invasion, pT3b) was diagnosed with cutaneous, lymph nodes, and multiple bone metastases. The patient was in good clinical conditions but complained of severe back pain. The histological diagnosis of the node biopsy in the right axillary node was melanoma metastasis with a *V600 BRAF* mutation (the specific aminoacidic substitution was not identified). In April 2019, the patient started target therapy with dabrafenib (150 TD) and trametinib (2 mg OD). A fractioned RT was performed to the tract L2, L3, and L4 (total dose 2000 cGy, delivered in 5 daily fractions). The subsequent 18FDG PET/CT scan showed a complete metabolic response. However, after 3 months, the patient complained of severe pains in the thoracic and pelvic region. The 18FDG PET/TC scan showed bone recurrences at the level of the sternum, costs, right homer, right iliac bone, and left ischiopubic branch. A new cycle of fractioned RT was performed directed to the sternum (total dose 3000 cGy, in 5 daily fractions) and the right sacroiliac joint (total dose 2000 cGy, in 5 daily fractions). The subsequent 18FDG PET/TC showed a metabolic progression of the humeral metastasis; therefore, fractioned RT was also administered at this site (total dose 2000 cGy, in 5 daily fractions). During RT, the combination of dabrafenib/trametinib was continued at the same dose without relevant AEs.

## 3. Discussion

In recent years, the treatment strategy for melanoma patients with *BRAF* mutation has improved with the introduction of effective treatments. Surgical resection of primary melanoma is commonly performed, and there is an opportunity to offer adjuvant therapy for patients with a high risk of relapse. In metastatic settings, several treatments are available, including immunotherapy and target therapy. In particular, systemic therapies have been significantly enriched with the approval of agents directed against the mitogen-activated protein kinase (MAPK) pathway [20]. This pathway consists of different proteins involved in signal transduction, leading to cellular growth and proliferation, which are important for cancer development and represent a therapeutic target in several malignancies [21]. In melanoma, the first approved drugs directed against the MAPK pathway were BRAFi, which first showed efficacy as single agents, improving the response rate (RR), progression-free survival (PFS), and overall survival (OS) when compared to chemotherapy in the metastatic setting [22,23]. However, an important mechanism of resistance to BRAF inhibition is the downstream reactivation of the MAPK pathway [24,25]. This event is also responsible for the increased risk of developing skin tumors during therapy with BRAFi [26,27]. Inhibition of the downstream MEK protein is one of the mechanisms to overcome resistance to BRAFi and to reduce the incidence of AEs occurring during BRAFi treatment. In fact, the simultaneous use of BRAFi and MEKi inhibitors has led to increased survival rates and a reduced risk of second tumors [10].

In the metastatic setting, the combination of different BRAFi and MEKi increased the median PFS in different phase III clinical trials, showing an RR of around 70% and an OS of over 2 years [28,29,30,31,32,33,34]. More precisely, in COMBI-d, 423 patients with advanced melanomas harboring *BRAF* mutations (*V600E/V600K*) were treated with BRAFi dabrafenib plus MEKi trametinib or dabrafenib + placebo (PBO) [31]. The study reached both its primary endpoint, improved PFS (HR 0.67; 95%CI 0.53–0.84), and the secondary endpoints: OS (HR 0.7; 95%CI 0.55–0.92) and RR (69% vs. 53%). In the COMBI-v trial, the combination dabrafenib + trametinib was superior to the BRAFi vemurafenib alone [35]. 704 advanced melanoma patients with *V600E/V600K BRAF* mutations were included. Dabrafenib/trametinib allowed better results in terms of OS (HR 0.6; 95%CI 0.53–0.89), PFS (HR 0.56, 95%CI 0.46–0.69) and RR (64% vs. 51%). Similar results were obtained with other combinations, such as vemurafenib (BRAFi) and cobimetinib (MEKi) in the CO-BRIM trial [28], and encorafenib (BRAFi) + binimetinib (MEKi) in the COLUMBUS study [36]. TT has also shown efficacy in the adjuvant setting. In the COMBI-AD trial [37], 870 patients with resected stage IIIA-D V600E/V600K BRAF-mutant melanoma were randomized to dabrafenib + trametinib versus a placebo for 12 months. The study met its primary endpoint, showing improved relapse-free survival (RFS) (HR 0.47, 95%CI 0.39–0.58).

In a retrospective study on 95 progressing patients, treatment beyond progression-TBP- (BRAF inhibitors with or without MEKi) in 37 patients resulted in a better PFS as compared to the other patients (*n* = 58) starting a new different line of therapy and/or supportive care (6.9 versus 3.8, *p* < 0.001) and in a better OS (11.6 versus 2.0 months, *p* < 0.001) [38]. Interestingly, normal lactate dehydrogenase levels, lower disease burden, and brain metastasis were linked to improved OS in patients undergoing TBP. In contrast, in a multicenter retrospective analysis carried out in 180 melanoma metastatic patients in progression with TT, the continuation of BRAF inhibitors did not impact OS, while immunotherapy with anti-PD1 drugs significantly improved OS [39]. In another recent study, 70 patients with BRAF-mutated advanced melanoma in progression on Vemurafenib treatment were divided into two groups: 35 who changed therapy and 35 who continued vemurafenib. Better PFS and OS were reported in TBP patients (PFS 5.6 months versus 4.0 and OS 12.8 months versus 6.3 months) [40].

The contemporary administration of RT and TT is usually not allowed in clinical trials due to the potential increased risk of BRAFi radio-sensitization [41,42]. However, in the metastatic setting, RT is often used because melanoma cells present a large spectrum of radio-sensitivity [43]. SRT and WB-RT are employed in the treatment of brain metastases, and fractioned or high-dose regimens are administered for pain control (i.e., symptomatic bone metastases) or to treat single metastases in oligoprogressive disease [44,45,46]. So far, the decision to interrupt or continue TT while administering RT has been debated. In fact, some retrospective reports on the contemporary administration of BRAFi and RT describe a high rate of AEs (i.e., dermatitis), in particular with high-dose RT regimens [41,42]. On the other hand, acceptable tolerability with the co-administration of BRAFi and fractioned regimens, such as whole-brain radiotherapy (WB-RT) or SRT, is reported. [47,48]. It is known that BRAFi has the potential for radio-sensitization [41]. This effect could enhance tumor control due to the reduction of radio-resistance depending on BRAF mutations [41,49]. In a multicentric analysis conducted by Hecht, 161 melanoma patients treated with concomitant BRAFi and RT were included [41]. An increased incidence of acute radiodermatitis ≥ grade 2 was reported in patients treated with conventionally fractioned RT (but not SRT) and concomitant BRAFi. Among the different BRAFi, radiodermatitis was more frequent with vemurafenib (40%) than with dabrafenib (26%), probably due to the different binding affinity of the two agents. Indeed, vemurafenib is able to bind both mutant BRAF and other RAF kinases, differing from dabrafenib, which is more selective for mutant BRAF [41,50]. Furthermore, it should be taken into account that vemurafenib is commonly administered at the maximum tolerated dose (MTD), whereas the standard dose of dabrafenib is lower than that of MTD [50]. In another large retrospective multicentric study, Hecht et al. analyzed 155 BRAF-mutant melanoma patients treated with either vemurafenib (*n* = 110) or dabrafenib (*n* = 45) as single agents and RT (including WB-RT, SRT brain, bone metastases, axillary lymph nodes, mediastinal metastases, and soft tissue metastases) [51]. Better survival and safety outcomes were reached if vemurafenib was interrupted rather than continued during the administration of RT [51]. Regarding patients treated with dabrafenib, OS and PFS were similar between the groups that interrupted or continued the drug during radiotherapy. The most common toxicity was cutaneous, such as keratoacanthoma or SCC. In this regard, it is known that BRAFi can cause cutaneous adverse events, such as folliculitis or SCC; therefore, a rate of skin toxicity can be related to BRAFi alone. Mucosal adverse events (rarely of severe grade) in the upper gastro-intestinal tract, esophagus, and rectum, and some cases of pneumonitis were also reported. No significant increase in neurotoxicity was described. A unique case of fatal hepatic bleeding and rare lethal bleeding in the lung and brain was reported [14]. Considering these observations, the Eastern Cooperative Oncology Group (ECOG) suggests the interruption of BRAFi during RT, holding these drugs at least 3 days before and after RT administration in case of fractioned regimens and 1 day before and after in case of SRT [17].

In the case series described herein, the patients had a prolonged benefit with dabrafenib and trametinib without adverse events. Considering the limited progression, RT was performed to achieve local control in association with dabrafenib and trametinib employed beyond progression. RT doses were established according to anatomical site, volume, and previous treatments. Different metastatic sites were irradiated (bone, liver, brain, lymph nodes) without interruption of TT. None of the patients reported increased toxicity.

## 4. Conclusions

The combination of dabrafenib and trametinib and, more generally, BRAFi and MEKi, represents a valid option for patients affected by BRAF-mutant melanoma. The main limitation of the available studies on the contemporary administration of TT and RT is their retrospective nature. These reports described increased toxicity due to the radio-sensitizing effect of TT when RT is contemporarily administered. However, the same radio-sensitizing effect could result in a better clinical outcome. Moreover, different regimens could determine a different risk of developing cumulative AEs. Therefore, the interruption of drugs should be carefully discussed. The five patients described in this series did not develop any AEs while receiving RT without interrupting dabrafenib or trametinib. This case series strengthens the possibility of continuing the administration of TT during different RT regimens in selected patients. Therefore, a multidisciplinary discussion with a personalized approach to every patient should be chosen. When a patient obtains a long benefit from dabrafenib and trametinib without experiencing relevant AEs, and RT is considered a good option for the control of oligoprogressive disease, TT could be continued beyond progression, limiting the time of interruption during RT. Indeed, a long period of TT interruption during radiotherapy could lead to a progression disease in other sites, particularly in patients with a high tumor load. Of note, the site undergoing irradiation and the type of radiation should also be taken into consideration. During treatment with dabrafenib and trametinib, periodical instrumental assessments and physical examinations can detect an early progression of the disease, for which RT in addition to TT beyond progression can be carried out. Prospective studies are needed to explore the efficacy and safety of the contemporary administration of BRAF/MEK inhibitors and radiotherapy for oligo-progressive disease. Some trials are ongoing with this specific intent (NCT03818503, ClinicalTrials.gov, accessed on 29 December 2022).

## Figures and Tables

**Table 1:** Patients treated with RT and TT beyond progression.

Patient	Age	Site of Metastases at TT Initiation	Site of Progression	TTP (m)	RT (cGy)	AEs
1	70	retro-mandibular and latero-cervical lymph-nodes; right hypogastric mesenteric tissue	retro-mandibular and latero-cervical lymph-nodes	20	retro-mandibular (2000); latero-cervical (2000)	none
2	70	lung, abdominal lymph-nodes; adrenal glands	brain	6	SRT on 3 brain metastases (2550 per lesion)	none
3	65	axillary lymph-node, subcutaneous lesions, liver, lung	liver	9	Liver S6-7 (3000)	none
4	47	lung	brain	8	Whole brain (1200)	none
5	52	skin, lymph-nodes, bone	bone	3	sternum (3000); sacroiliac region (2000), homer (2000)	none

TT: target therapy; m: months; RT: radiotherapy; SRT: stereotactic radiotherapy AEs: adverse events (related to administration of RT and TT); TTP: time to progression; S: segment.

## Data Availability

Not applicable.
